# *Bradyrhizobium* as the Only Rhizobial Inhabitant of Mung Bean (*Vigna radiata*) Nodules in Tropical Soils: A Strategy Based on Microbiome for Improving Biological Nitrogen Fixation Using Bio-Products

**DOI:** 10.3389/fpls.2020.602645

**Published:** 2021-01-12

**Authors:** Vinício Oliosi Favero, Rita Hilário Carvalho, Victória Monteiro Motta, Ana Beatriz Carneiro Leite, Marcia Reed Rodrigues Coelho, Gustavo Ribeiro Xavier, Norma Gouvêa Rumjanek, Segundo Urquiaga

**Affiliations:** ^1^Universidade Federal Rural do Rio de Janeiro, Seropédica, Brazil; ^2^Embrapa Agrobiology, Seropédica, Rio de Janeiro, Brazil

**Keywords:** mung bean, microbiome, nodule, native rhizobia, *Bradyrhizobium*, symbionts, *Pseudomonas*, biological nitrogen fixation

## Abstract

The mung bean has a great potential under tropical conditions given its high content of grain protein. Additionally, its ability to benefit from biological nitrogen fixation (BNF) through association with native rhizobia inhabiting nodule microbiome provides most of the nitrogen independence on fertilizers. Soil microbial communities which are influenced by biogeographical factors and soil properties, represent a source of rhizobacteria capable of stimulating plant growth. The objective of this study is to support selection of beneficial bacteria that form positive interactions with mung bean plants cultivated in tropical soils, as part of a seed inoculation program for increasing grain yield based on the BNF and other mechanisms. Two mung bean genotypes (Camaleão and Esmeralda) were cultivated in 10 soil samples. Nodule microbiome was characterized by next-generation sequencing using Illumina MiSeq 16S rRNA. More than 99% of nodule sequences showed similarity with *Bradyrhizobium* genus, the only rhizobial present in nodules in our study. Higher bacterial diversity of soil samples collected in agribusiness areas (MW_MT-I, II or III) was associated with Esmeralda genotype, while an organic agroecosystem soil sample (SE_RJ-V) showed the highest bacterial diversity independent of genotype. Furthermore, OTUs close to *Bradyrhizobium elkanii* have dominated in all soil samples, except in the sample from the organic agroecosystem, where just *B. japonicum* was present. Bacterial community of mung bean nodules is mainly influenced by soil pH, K, Ca, and P. Besides a difference on nodule colonization by OTU sequences close to the *Pseudomonas* genus regarding the two genotypes was detected too. Although representing a small rate, around 0.1% of the total, *Pseudomonas* OTUs were only retrieved from nodules of Esmeralda genotype, suggesting a different trait regarding specificity between macro- and micro-symbionts. The microbiome analysis will guide the next steps in the development of an inoculant for mung bean aiming to promote plant growth and grain yield, composed either by an efficient *Bradyrhizobium* strain on its own or co-inoculated with a *Pseudomonas* strain. Considering the results achieved, the assessment of microbial ecology parameters is a potent coadjuvant capable to accelerate the inoculant development process and to improve the benefits to the crop by soil microorganisms.

## Introduction

Mung bean (*Vigna radiata* (L.) Wilczek) is a widely cultivated crop on the Asian continent. It has good adaptability to tropical climate conditions and its grain has high nutritional value (Du et al., 2018; Yi-Shen et al., 2018). Mung bean was introduced in Brazil several decades ago, being characterized as a small-scale crop with low national consumption (Duque et al., 1987; Barradas et al., 1989). However, the cultivated area has been increasing in recent years, aiming to meet the export demand of Asian countries, especially India. Nowadays, mung bean has been cultivated in agribusiness areas of the Brazilian Cerrado in succession to soybean and corn. The low implantation cost, short cycle, good temperature adaptation and water regime contribute to its development and grain yield increasing its acceptance by farmers (Nair et al., 2012; Hanumantharao et al., 2016; Sharma et al., 2016).

Mung bean benefits from biological nitrogen fixation (BNF) through association with native rhizobia (Herridge et al., 2005), which decreases the demand for the nitrogen fertilizer application. Overall, mung bean is known for their low symbiotic specificity with soil rhizobia native (Yang et al., 2008; Zhang et al., 2008), even among species of the *Bradyrhizobium* genus (Risal et al., 2012) and it benefits from seed inoculation with efficient selected elite strains (Delić et al., 2011). Phylogeny and symbiotic efficiency studies show that organisms belonging to the *Bradyrhizobium* genus are the most important micro-symbiont for this species (Yang et al., 2008; Zhang et al., 2008; Appunu et al., 2009; Risal et al., 2012). In addition to *Bradyrhizobium*, other genera belonging to the large group of rhizobia are also reported as mung bean symbionts, despite being less studied: *Ensifer* (= *Sinorhizobium*) (Hakim et al., 2018, 2020), *Rhizobium* (Yang et al., 2008; Zhang et al., 2008), and *Mesorhizobium* (Lu et al., 2009).

Sequencing 16S rRNA gene amplicons from root nodules, Hakim et al. (2018) showed a codominance of *Bradyrhizobium* and *Ensifer* genera as micro-symbionts of mung bean cultivated in Pakistan. However, a subsequent study identified a dominance of up to 94 and 99% of sequences belonging to *Bradyrhizobium* and *Ensifer* genera, respectively, depending on the soil (Hakim et al., 2020). This difference in the mung bean nodule microbiome shows that soil characteristics directly influence the plant-microorganism relationship.

Studies on microbial community of legume root nodules have shown the presence of several non-rhizobial bacteria (NRB) genera (Leite et al., 2009, 2017; Martínez-Hidalgo and Hirsch, 2017; Hakim et al., 2018). Until recently, it was thought that legume nodules were only inhabited by rhizobia, according to the plant trait regarding host specificity. However, recent studies have shown a different picture where nodules from several legume species have a great diversity of microorganisms (Aserse et al., 2013; De Meyer et al., 2015; Leite et al., 2017; Martínez-Hidalgo and Hirsch, 2017; Trabelsi et al., 2017; Cardoso et al., 2018; Zhang et al., 2018). Hence, the microbial community structure and the role of most NRB in the plant/rhizobia symbiotic relationship are still poorly understood. The evaluation of nodule microbial community composition is a potent tool which enables selecting beneficial microorganisms to improve plant development. Furthermore, this knowledge can assist in the development of multi-organism biological products aiming to increase grain yield. Several studies have focused on the possible benefits to cultivated plant by atmospheric N fixation (De Meyer et al., 2015; Andrews and Andrews, 2017), biocontrol activity (Berg et al., 2017), or plant growth promotion (Tariq et al., 2014), among others.

There are currently no studies related to BNF and nodule NRB for mung bean under agroecosystem conditions in Brazil. Therefore, we used the 16S rRNA Illumina MiSeq sequencing to investigate bacterial mung bean root nodule microbiomes, aiming to characterize the composition of rhizobia and NRB in different soils in Brazil, and to verify differences in the nodule communities between two mung bean genotypes. Our study aims to support the selection of beneficial microorganisms for mung bean plants as part of a seed inoculation program for increasing grain yield based on BNF and other bacterial functions.

## Materials and Methods

### Plant Cultivation

**FIGURE 1:**
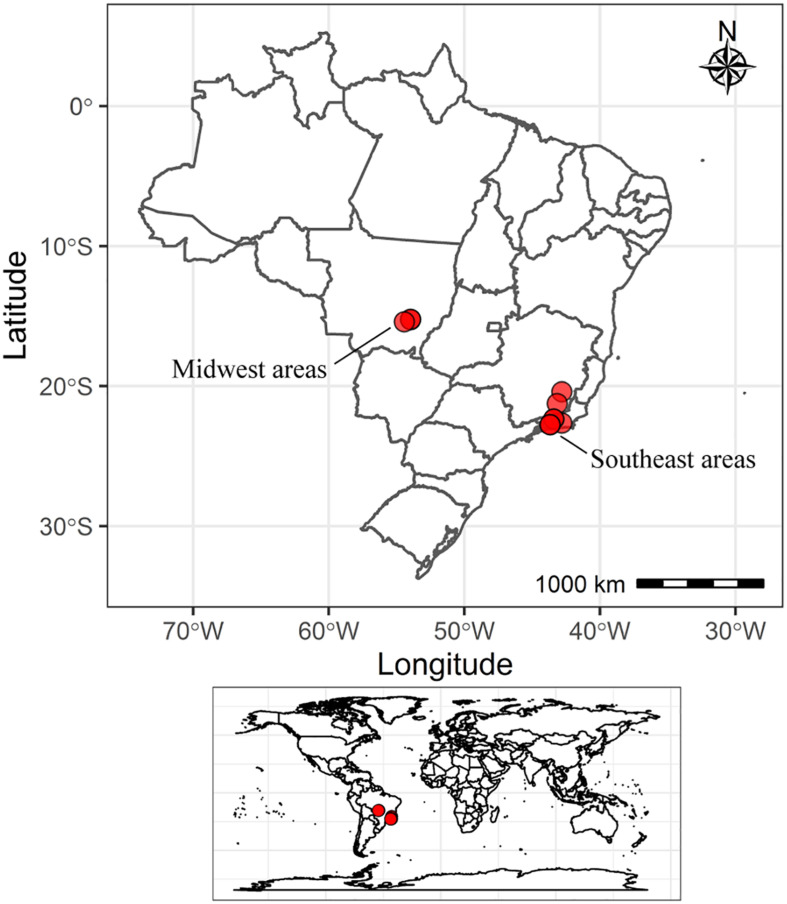
Map of Brazil with the locations of soil sample collection sites.

Mung bean plants were grown using Leonard jars (Vincent, 1970) maintained in a greenhouse located in Seropédica, RJ, Brazil. An experiment was conducted in a factorial scheme (soil × genotype) and a randomized block design with three replications: 10 soil samples from different regions of Brazil and two mung bean genotypes, MGS Esmeralda (Vieira et al., 2008) and the Camaleão cultivars. Ten soil samples were collected in agricultural areas located in the Midwest and Southeast Brazilian regions, previously cultivated with mung bean and/or other legumes (Figure 1 and Table 1). Ten simple samples were collected with an auger at 0–20 cm depth, homogenized and sieved (<4 mm) to obtain a composite sample. Two mung bean cultivars registered at the Brazilian Ministry of Agriculture, Livestock and Supply were used: MGS Esmeralda (registry 22096) and Camaleão (registry 36829). MGS Esmeralda was developed by Asian Vegetable Research and Development Center (Shanhua, Taiwan), as a result of crossing between the lines VC 1973A and VC 2768A (Vieira et al., 2008). Camaleão is a recently released cultivar in 2018, developed by Minas Gerais Agricultural Research Agency, EPAMIG. Seeds of both cultivars are available at EPAMIG (asagro@epamig.br).

**TABLE 1:** Soil sample identification, location, cultivation history, precipitation, and fertility analysis of soil material used for planting mung bean in Leonard jars.

Soil sample*	Latitude and longitude	Cultivation history	Annual average rainfall**	pH	Al^3+^	Ca^2+^	Mg^2+^	P	K^+^	C
				
			mm		cmol_c_ dm^–3^			mg L^–1^		%
MW_MT-I	15°14′05.8″S 53°58′51.1″W	Soybean, corn and sorghum	1,794	6.45	0.00	3.50	1.56	104.37	136.98	1.35
MW_MT-II	15°13′37.9″S 53°58′48.1″W	Mung bean, soybean, cowpea and corn	1,794	5.55	0.00	2.95	0.92	115.33	270.40	1.40
MW_MT-III	15°23′33.5″S 54°26’46.7”W	Mung bean, soybean, cowpea and corn	1,794	4.30	0.34	2.88	0.77	214.78	190.34	1.85
SE_MG-I	20°24′07.57″S 42°49′05.08″W	Mung bean	1,269	4.61	0.33	1.24	0.36	74.47	149.39	0.70
SE_MG-II	21°14′36.74″S 43° 9’30.55”W	Common bean	1,391	5.98	0.00	4.04	1.03	71.09	216.39	1.31
SE_RJ-I	22°38′4.61″S 42°48′40.40″W	Mung bean, common bean and cowpea	1,100	4.44	2.35	1.29	0.39	138.17	49.00	3.64
SE_RJ-II	22°20′52.36″S 43°25′2.24″W	Common bean and cowpea	1,192	5.74	0.00	1.70	0.24	92.15	80.42	0.44
SE_RJ-III	22°20′54.84″S 43°25′2.27″W	Azuki and mung bean	1,192	5.91	0.00	3.32	1.26	161.48	224.70	1.27
SE_RJ-IV	22°45′22.27″S 43°40′2.03″W	Common bean and cowpea	1,100	5.73	0.00	1.90	0.49	24.03	85.89	0.68
SE_RJ-V	22°45′16.36″S 43°40′28.04″W	Mung bean, soybean, peanut and other vegetables	1,100	6.51	0.00	3.40	0.73	155.59	139.96	0.86

*Soil origin: SE, Southeast; MW, Midwest; RJ, Rio de Janeiro; MG, Minas Gerais; MT, Mato Grosso. **Annual average rainfall for the region based on data from the last 10 years (Source: National Institute of Meteorology – INMET). Al^+3^, aluminum; Ca^2+^, calcium; Mg^2+^, magnesium; P, phosphorus; K^+^, potassium; C, carbon.

Seven and three soil samples were collected from areas under conventional and organic management, respectively. The three Midwest areas (MW_MT-I, MW-MT-II, and MW_MT-III), the two Minas Gerais state areas (SE_MG-I and SE_MG-II) and two areas from Rio de Janeiro state (SE_RJ-I and SE_RJ-IV) were under conventional management, while the three areas under organic management were located in Rio de Janeiro state (SE_RJ-II, SE_RJ-III, and SE_RJ-V). Six areas belong to experimental fields located in research and educational governmental centers used for field trials with different agricultural crops (SE_MG-I, SE_MG-II, SE_RJ-II, SE_RJ-III, SE_RJ-IV, and SE_RJ-V). The Midwest areas are typical of intensive agriculture located in the Brazilian Cerrado region (MW_MT-I, MW-MT-II, and MW_MT-III), while SE_RJ-I is characterized as a family farming.

Leonard jars were adapted so that the soil was used as an inoculum. The vessels were filled with approximately 600 cm^3^ of substrate composed of sterilized gravel and vermiculite (2:1 v v^–1^). A layer of soil material (100 cm^3^) was added on the surface of the sterilized substrate according to diagram on Supplementary Figure S1. Seeds were sown directly in the soil layer and then a final layer of sterilized sand was added to the top. The seeds used were superficially disinfested by immersion in 70% ethanol and hydrogen peroxide for one and three minutes, respectively, followed by 10 washes in sterile distilled water (Vincent, 1970).

Five seeds per jar were sown and then thinned to two plants per jar. Next, 300 mL of Norris’ nutrient solution devoid of N and sterilized in an autoclave was applied, weekly into each jar (Norris and Date, 1976). In the first week, a nutrient solution with half the ionic strength was used. Plants were collected at 35 days after emergence.

### DNA Extraction From Nodules

Plants were collected, nodules were detached from the roots and kept in a super freezer (−80°C). For extraction, nodules were superficially disinfested by soaking in 70% ethanol for one minute and in sodium hypochlorite (4–6%) for five minutes, followed by eight washes in sterile distilled water. The disinfestation procedure was performed in 15 mL tubes using approximately 2 mL of reagent or water at each step added and removed by an automatic pipette. Homogenization was carried out in a bench vortex for 15 s in each step. After disinfestation, 500 mg of nodules were macerated in liquid nitrogen, followed by DNA extraction using the Fast DNA Spin Kit for Soil (MObio) according to the manufacturer’s instructions.

### 16S rRNA Gene Amplification

Nodule DNA was purified and subjected to next-generation sequencing (NGS) with Illumina MiSeq library preparation by Macrogen (Korea, Seoul). The V3–V4 region of the 16S rDNA gene was amplified using 341F (CCTACGGGNGGCWGCAG) and 805R (GACTACHVGGTATCTAATCC) (Herlemann et al., 2011) primers with amplification of approximately 440 bp.

### Bioinformatics and Data Analysis

The sequences were analyzed with Mothur software (v. 1.44.2) (Schloss et al., 2009). The forward and reverse sequences were grouped into contigs using the make.contigs command and processed using screem.seqs to remove ambiguous sequences and those that had more than eight homopolymers. Sequences under 440 and over 443 base pairs (bp) were excluded. They were then aligned using the Silva ribosomal RNA gene database (v. 138) (Quast et al., 2013). Misaligned strings and non-informative columns were removed using the screen.seqs and filter.seqs commands. Rare sequences were grouped with the abundant sequences using the pre.cluster command with a difference threshold of 4 bp. Chimeric sequences were then removed using the chimera.vsearch command (Rognes et al., 2016). Classification was performed using the Ribosomal Database Project (Cole et al., 2009) to gender level, with 80% bootstrap. Mitochondria, chloroplast, archaea, eukaryote, and unknown domain sequences were eliminated using the remove.lineage command. Single sequence operational taxonomic units (OTUs) were removed and samples were randomly subsampled to the smallest sample size. Taxonomy and distribution data from OTUs were exported and used in other programs. We filter OTUs based on abundance, and OTUs with less than 40 sequences have been removed.

Rarefaction curves were generated by Mothur and plotting in the R environment (R Core Team, 2020). Number of observed OTUs, Chao1 estimator, diversity by the Shannon index and Shannon evenness were also generated by Mothur, and analyzed for significant differences through analysis of variance (anava) in the R environment (v. 4.0.2) (R Core Team, 2020) using the ExpDes.pt package (v. 1.2.0) (Ferreira et al., 2013). Beta diversity data were generated using the phyloseq package (v. 1.32.0) (McMurdie and Holmes, 2013), and analyzed for significant difference through permutational multivariate analysis of variance (PERMANOVA) using the “adonis” function and for dispersion of variances with the “betadisper,” both with 999 permutations and using the vegan package (v. 2.5-6) (Oksanen et al., 2019) in the R environment (R Core Team, 2020). For plotting, we use the ggplot2 (v. 3.3.0) (Wickham, 2016) and cowplot (v. 1.0.0) (Wilke, 2016) packages.

After filtering the less abundant OTUs, we carry out an alignment and phylogenetic analyze of the most abundant sequences of the classified OTUs. For this, we use the MEGA 7 software (Kumar et al., 2016). The phylogenetic tree was built using the maximum likelihood (ML) method using the Kimura 2-parameter + G model (Kimura, 1980). This model was chosen based on the best model tool available in the MEGA 7 software. The bootstrap values were shown when the relationships represented were observed in at least 50% of the 1,000 replicates.

## Results

### Characteristics of Amplicon Libraries

A total of 4,301,345 raw tags stemming from 16S rRNA gene amplification were obtained. After read-quality filtering and perform a 97% sequence similarity using Mothur with Silva ribosomal RNA gene database, 1,182,393 sequences were acquired, corresponding to 19,706 sequences per sample (range = 19,559 to 19,777), spread along 1, 094 OTUs.

Next, a second sequence filtering aiming at eliminating low abundance and low variance OTUs yields 1,177,815 sequences and a mean value per sample of 19,630 (range = 19,451 to 19,727). This last step removed 4,578 sequences, approximately 0.39% from the total, but brought down OTU number to 17, reflecting a high level of rare sequences scattered through the samples obtained from the original data. The 17 OTUs range from a minimum of 42 to a maximum of 1,175,646 sequences. On average 10 ± 2 OTUs were detected per sample.

**FIGURE 2:**
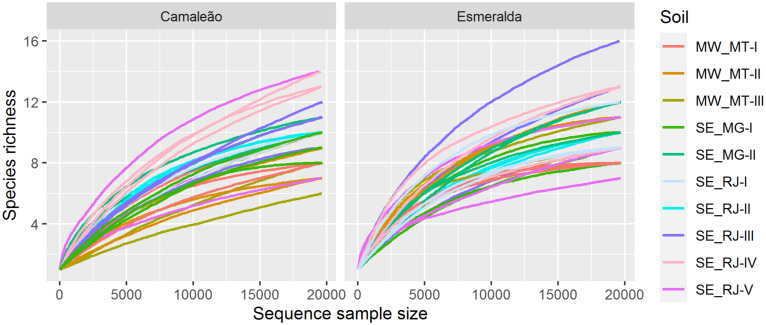
Rarefaction curves for species richness at 97% similarity as a function of sample size for Camaleão and Esmeralda mung bean genotypes and 10 soil samples.

Besides the high rare sequence amount, a unique OTU present in all samples accounted for more than 99.8% suggesting a framework of ecological dominance on nodule microbiome. The methodology used for filtering our data produced satisfactory rarefaction curves (Figure 2) and estimated coverage values greater than 99.9% (Good, 1953), which revealed that OTU libraries were sufficiently large to capture the most common OTUs and, that the probability to find a new OTU is nearly zero (coverage deficit).

### Bacterial Community Richness and Diversity

**FIGURE 3:**
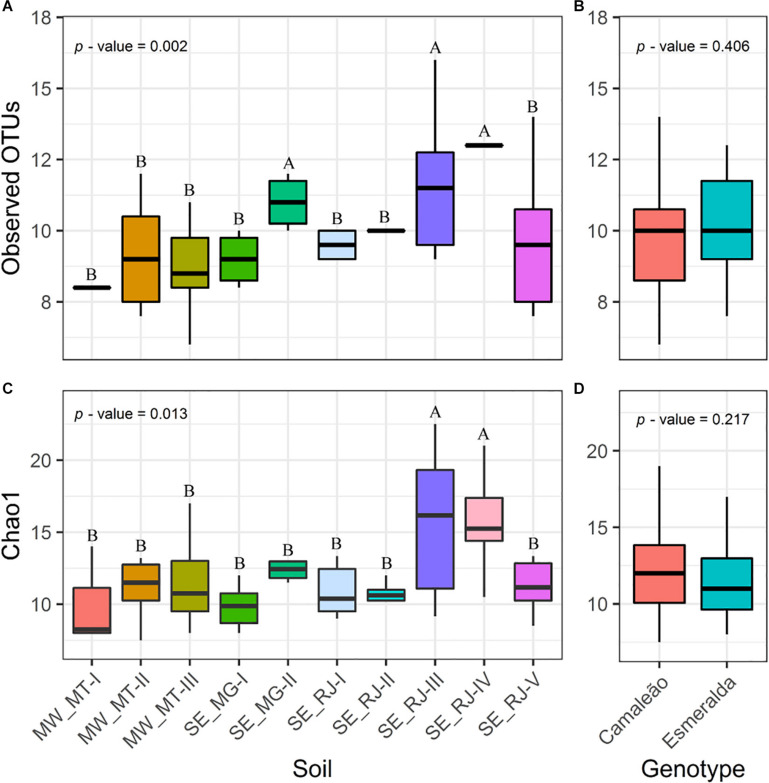
**(A,B)** Number of observed OTUs and **(C,D)** estimated richness by Chao1 of bacterial communities from nodules of Camaleão and Esmeralda mung bean genotypes inoculated with 10 different soils. *p*-values are based on anava. Distinctive letters indicate statistical difference by the Scott–Knott test at 5% probability.

There was not a significant interaction between soil samples and mung bean genotypes for number of OTUs and richness index Chao1 (Figure 3). A greater number of OTUs was recovered from nodules of mung bean cultivated on sterile substrate mixed with soil samples collected at SE_MG-II, SE_RJ-III, and SE_RJ-IV in comparison to other soil samples (*p* = 0.002) (Figure 3A). SE_RJ-III and SE_RJ-IV soil samples also promoted high values for Chao1 index (*p* = 0.013) (Figure 3C). Mung bean genotypes on its own were not capable to influence observed OTUs and Chao1 index (Figures 3B,D).

**FIGURE 4:**
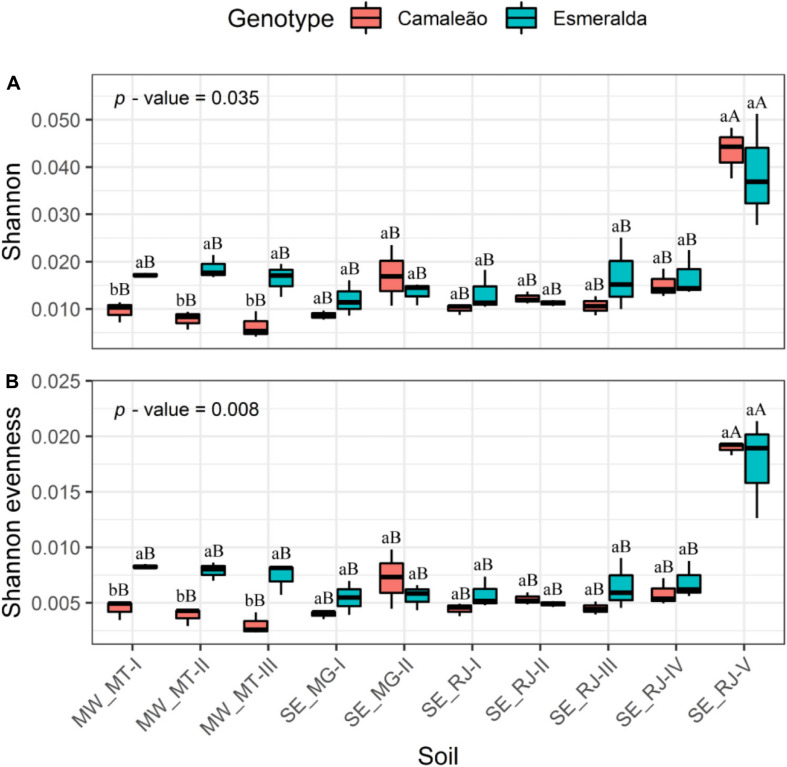
**(A)** Shannon’s diversity and **(B)** evenness of bacterial communities from nodules of Camaleão and Esmeralda mung bean genotypes inoculated with 10 different soils. *p*-values are based on anava. Distinctive letters, lowercase between genotypes and uppercase between soils, indicate statistical difference by the Scott–Knott test at 5% probability.

Community Shannon’s diversity and evenness showed interaction between mung bean genotypes and the origin of soil samples, at a probability of 0.035 and 0.008, respectively (Figures 4A,B). Nodule microbiome from plants grown on MW-MT-I, MW-MT-II, and MW-MT-III soil samples revealed values for Esmeralda genotype that were about twice the values observed for Camaleão considering both diversity and evenness. Diversity of remaining genotypes were not affected by soil origin.

Soil sample from SE_RJ-V area showed higher diversity and evenness indices when compared to other soil samples (Figures 4A,B). This area is an organically managed area where diverse plant cultivation practice may be associated to high nodule microbiome diversity, although it is not capable to stimulate a high OTU number nor a Chao1 richness index (Figures 3A,C). Diversity and evenness indices deal with both diversity and abundance, but according to our data the result is related mainly to abundance.

### Mung Bean Genotypes and Soil Origins on Nodule Bacterial Community Composition

**FIGURE 5:**
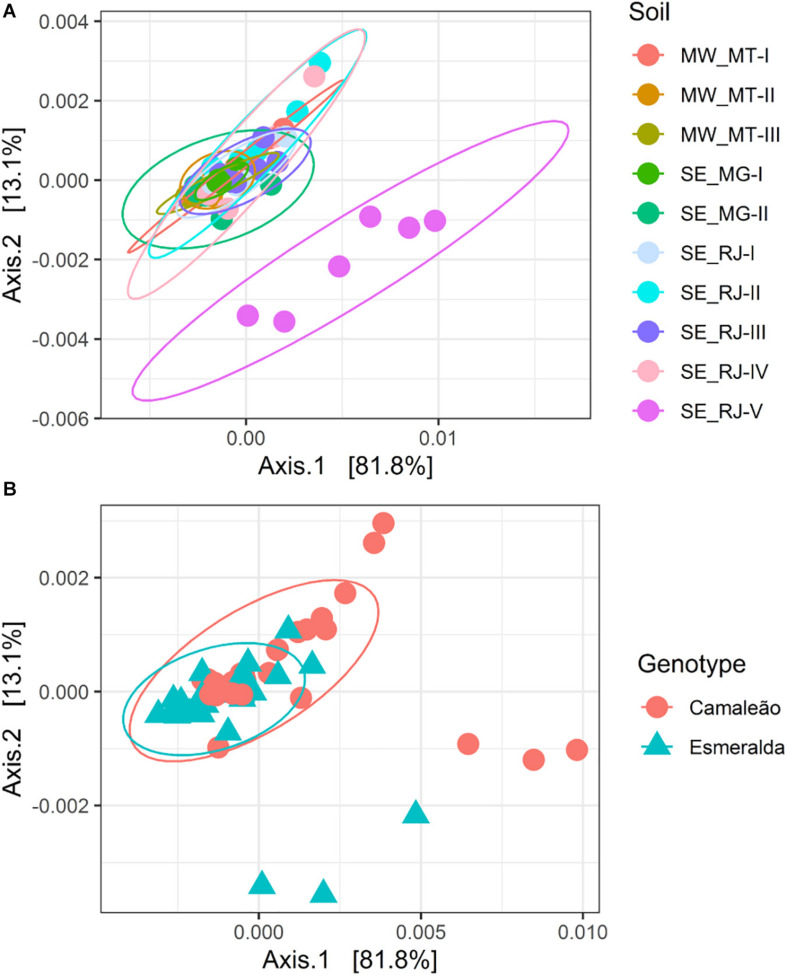
Principal coordinates analysis (PCoA) of Bray-Curtis distances and the Permutational MANOVA (PERMANOVA) according to nodule bacterialcommunities estimated by 16S rRNA gene sequencing. **(A)** Samples coded for 10 soil samples (*p* = 0.001), and **(B)** from genotypes Camaleão and Esmeralda of *Vigna radiata* (*p* = 0.002) at the OTU level.

The beta diversity evaluation through PERMANOVA analysis showed a significant difference among soils (*p* = 0.001) (Figure 5A) and between genotypes (*p* = 0.002) (Figure 5B). Principal coordinate analysis (PCoA) explains a large part of the data variability, corresponding to 94.9% in the two axes. Regardless of the soil type, distribution of OTUs was homogeneous, except for nodule communities retrieved from SE_RJ-V soil sample, which was completely separated from the others (*p* = 0.001) (Figure 5A). Although Esmeralda genotype had a larger diversity than Camaleão, considering OTUs composition, soil origin influences the latter more than the former as it moves away from the central core, especially when cultivated in SE_RJ-V soil sample. Ten Camaleão bacterial communities are outside the intersection area, where most samples are concentrated. In contrast, just five Esmeralda bacterial communities are found outside the central core. Under these study conditions, both plant genotypes and soil origin seem to affect bacterial community diversity inhabitanting mung bean nodule.

**FIGURE 6:**
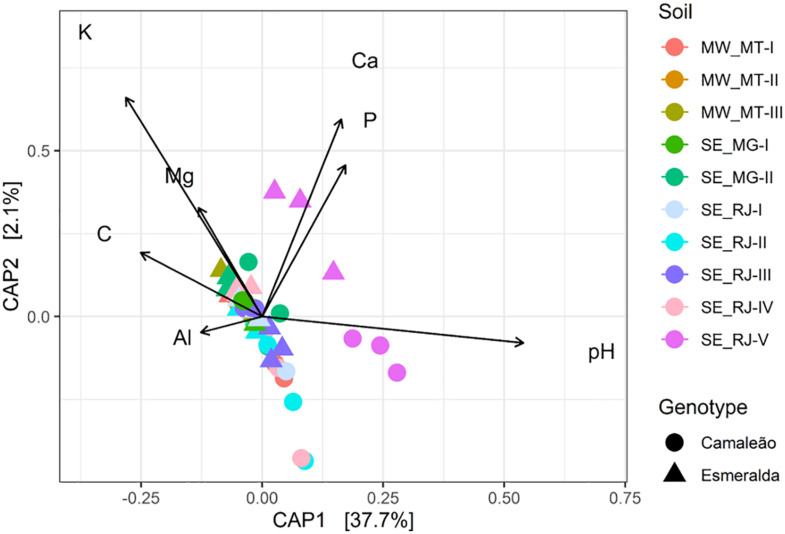
Canonical analysis of principal coordinates (CAP) of Bray-Curtis distances and the Permutational ANOVA between nodule bacterial communities (10 soil samples × 2 mung bean genotypes) estimated by 16S rRNA gene sequencing and the chemical data of soil samples presented in Table 1: pH, Al^3+^ (aluminum), Ca^2+^ (calcium), Mg^2+^ (magnesium), P (phosphorus), K^+^ (potassium), and C (carbon).

A Canonical analysis of principal coordinates (CAP) between nodule bacterial communities and chemical fertility variables of soil samples explained 39.8% of the variance considering the two axes, and an anova analysis determined the significance level (*p* = 0.001) (Figure 6). From this analysis, pH had a greater influence on the bacterial community of mung bean nodules from Camaleão genotype when grown in SE_RJ-V soil sample, whereas, Esmeralda nodule community in this soil sample is related to K, Ca, and P concentrations (Figure 6). In general, the points referring to the SE_RJ-V soil are more distant from the others, and presented a higher pH value, around 6.51, while the other soils, an average of 5.41.

### Characterization of Bacterial Taxa Present in Mung Bean Nodules and Phylogenetic Analysis

**FIGURE 7:**
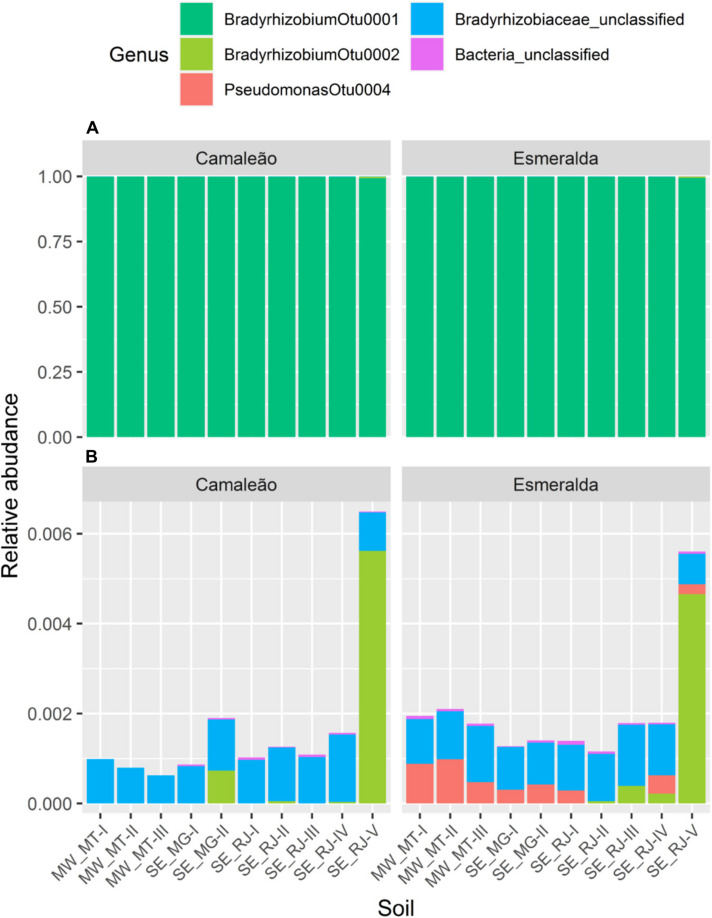
Relative abundance of sequences based on the 16S rRNA gene from nodules of Camaleão and Esmeralda mung bean genotypes cultivated in 10 different soil samples: **(A)** relative abundance for all OTUs; and **(B)** for low abundance OTUs, after removal of OTU0001 (*Bradyrhizobium*).

Upon filtration, the representative OTU_*S*_ retrieved from mung bean nodules belong only to *Alphaproteobacteria* and *Gammaproteobacteria* classes. The *Bradyrhizobium* genus was prevalent regardless of soil samples or mung bean genotypes, and two OTUs (OTU0001 and OTU0002) were associated to this genus (Figure 7A). OTU0001 corresponded to more than 99% of the sequences of nodule bacteria for all samples. Therefore, for better visualization of less abundant groups, we also present the data of relative distribution without OTU0001 (Figure 7B). In this case, *Bradyrhizobium* OTU0002 was characteristic of soil sample SE_RJ-V for both genotypes. These data corroborate the differences already observed for the alpha and beta diversity analyses (Figures 4A,B, 5A). No sequences representing other rhizobial genera were found.

After filtering the less abundant OTUs, we found only one NRB in nodule communities. The OTU0004 was classified as a *Pseudomonas* and it was recovered only from Esmeralda genotype nodules cultivated in eight soil samples (Figure 7B). This OTU corresponded approximately to 0.1% of the total sequences analyzed for the Esmeralda genotype. Only nodules from SE_RJ-I and SE_RJ-II soils were not colonized by *Pseudomonas*. As pointed out before, these results suggest a difference in the specificity trait between mung bean genotypes, as only Esmeralda genotype allows nodule occupation by *Pseudomonas* strains. Moreover, the data shows differences in soil bacterial communities regarding the *Pseudomonas* genus, since it was not present in Esmeralda nodules from plants grown in 2 out of 10 evaluated soils.

**FIGURE 8:**
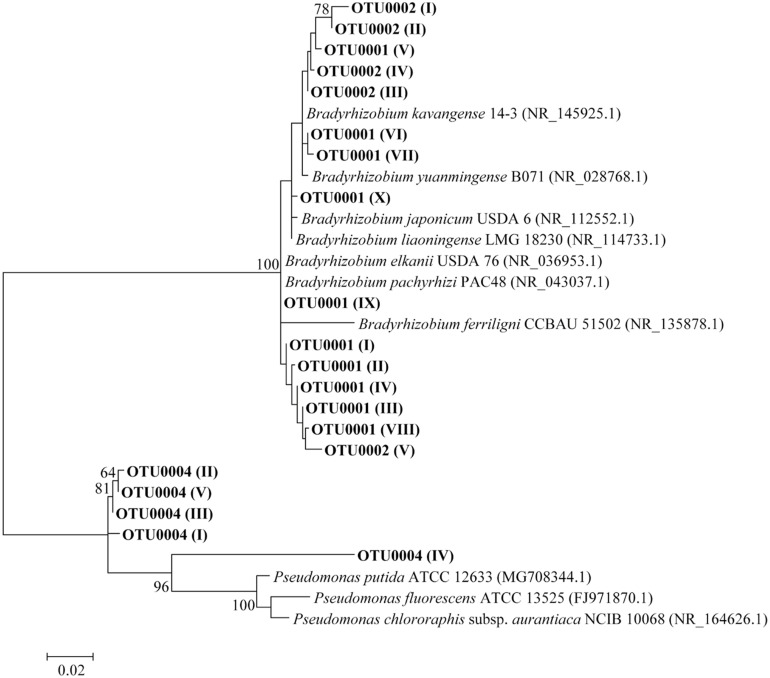
Maximum likelihood phylogenetic tree for OTUs classified, based on 16S rRNA gene sequences. Tree estimated through 441 base pair positions. The 10 most abundant sequences from OTU0001 and five from OTU0002 and OTU0004 were used. Letters in Roman numerals indicate order of abundance of the strings within their respective OTUs. Bootstrap values are shown when the relationships represented have been observed in at least 50% of 1,000 replicates. The tree was obtained using the Kimura 2-parameter + G model.

OTU0001 and OTU0002 related to *Bradyrhizobium* genus and OTU0004 related to *Pseudomonas* genus had 5,770, 587, and 111 representative sequences, respectively. We performed a phylogenetic analysis using the 10 most abundant sequences from OTU0001 and five from each OTU0002 and OTU0004, which represented 98.8% of the total sequences of our data after filtering. The other OTUs were not classified at the genus level, therefore, were not included. As for the representative sequences of *Pseudomonas*, none of the sequences were related to the described species (Figure 8).

**FIGURE 9:**
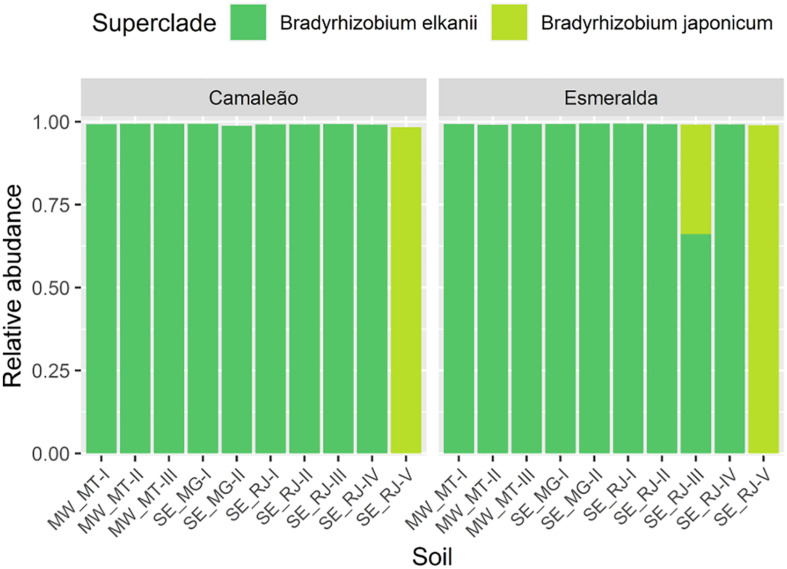
Distribution of the 10 most abundant sequences of OTU0001 and five of OTU0002 belonging to the *Bradyrhizobium* genus, based on the 16S rRNA gene from nodules of Camaleão and Esmeralda mung bean genotypes cultivated in 10 different soil samples.

The representative sequences of OTU0001 were divided between the *Bradyrhizobium japonicum* and *B*. *elkanii* superclades, while most of the OTU0002 sequences were grouped within *B*. *japonicum* (Figure 8). Besides that, all sequences associated to nodules from SE_RJ-V soil sample belong to *B*. *japonicum* (Figure 9), while, in the remaining soil samples, the vast majority of sequences is affiliated to *B*. *elkanii*. Furthermore, in this soil, all the sequences phylogenetically evaluated either from OTU0001 or OTU0002 were grouped with *B*. *japonicum*, regardless of the mung bean genotype (Figure 9).

## Discussion

As a general pattern, nodules from Esmeralda genotype presented greater bacterial diversity than nodules from Camaleão, especially when associated to certain soil samples tested in the present study. Domestication of plant species is recognized to cause a strong decrease on genetic diversity of modern crop cultivars (Pérez-Jaramillo et al., 2016). Therefore, germplasms submitted to an intense selection process through several crossing of varieties aiming to improve stability of a desirable trait tend to be more restrictive regarding microbial associations (Gepts, 2004; Mutch and Young, 2004; Gross and Olsen, 2010; Kim et al., 2014; Pérez-Jaramillo et al., 2016). Although plant traits capable to drive microbiome assembly and functions are largely unknown, plant breeding is understood as an opportunity to shape efficient colonization with elite strains, contributing to promote an increase in plant biomass and grain yield, or improve resistance to pests and disease (Partida-Martínez and Heil, 2011; Mendes et al., 2013; Berg et al., 2017). The low bacterial diversity determined on nodule microbiome of Camaleão genotype may suggest that as the breeding program proceeds, specificity toward the micro-symbionts increases and may be the reason why it is not colonized by *Pseudomonas* strains.

Considering soil origin influence, a greater alpha and beta bacterial diversity was found in nodules of plants cultivated in SE_RJ-V soil sample. A Canonical analysis of principal coordinates related the microbial community present in Camaleão nodules mainly to pH values, whereas Esmeralda nodule community is related to K, Ca, and P concentrations (Figure 6). It is known that soil microbial communities are influenced by soil properties (Fierer and Jackson, 2006; Santoyo et al., 2017). For example, a greater abundance of the *Ensifer* genus was found in mung bean nodules grown in higher pH soils (Hakim et al., 2020), and a positive relationship between microbial diversity with soil pH (Rousk et al., 2010; Andrew et al., 2012).

Besides that, plant genotype due to its physiological traits might interact in a specific way with soil attributes and that may result on distinct nodule microbial communities. Nevertheless, it must be emphasized that microbial communities from the remain nine soil samples did not differentiate between them (Figure 5). These data suggest that microbial community distribution is influenced by other factors than soil chemistry, such as the area crop history and management type. High crop diversity is a main feature associated to soil sample from SE_RJ-V area where organic agriculture follows the principle of plant diversification through crop rotation, green manure, crop alley and agroforestry as standard practices. SE_RJ-V area is an integrated system for agroecological production which has been implemented since 1993, aiming to establish a high diversity environment free from synthetic fertilizer and pesticide application attained by the constant cultivation of several and diverse green manure fertilizers, grain legumes, vegetable, and fruit species (Neves et al., 2005). Soils from organic farming systems are reported to have greater microbial diversity due to reduced use of agrochemicals (Ramirez et al., 2012; Hartmann et al., 2015; Weese et al., 2015; Xia et al., 2015; Wang et al., 2016, 2017). Furthermore, high diversity above ground tends to boost below ground diversity (Smalla et al., 2001; Van Der Putten et al., 2007; Berg and Smalla, 2009; Hartmann et al., 2009; Ofek et al., 2014), which may explain the highest diversity found in the nodule microbiome, since soil is regarded to be the main source of nodule bacteria (Marquez-Santacruz et al., 2010).

The observed prevalence of *B*. *japonicum* needs to be further investigated in this area, but it is possible that high diversity might also exert some influence on nodule microbiome. This high discrimation among the two superclades, brings a new knowledge about nodule bacteria and may be part of a strategy to improve BNF contribution for mung bean.

Nodule microbiome analysis of rhizobial populations shows the *Bradyrhizobium* genus as the predominant mung bean micro-symbiont in Brazilian tropical soils independent from both plant genotypes and whether soil samples present (or do not) a history of mung bean cultivation. A predominance of a rhizobial genus in legume nodules is commonly reported (Sharaf et al., 2019; Hakim et al., 2020; Rocha et al., 2020; Zheng et al., 2020). Isolation of diazotrophic strains from mung bean nodules cultivated in different soils worldwide have shown a predominance of *Bradyrhizobium* strains (Yang et al., 2008; Zhang et al., 2008; Appunu et al., 2009; Risal et al., 2012). However, *Ensifer* strains have also been reported to be isolated from mung bean nodules (Yang et al., 2008; Zhang et al., 2008; Pandya et al., 2013). In another study, a microbiome characterization of mung bean nodules from an experimental field area in Pakistan using pyrosequencing recently showed a co-dominance of strains from both *Bradyrhizobium* and *Ensifer* (Hakim et al., 2018). Illumina sequencing was used for analyzing nodule bacterial communities in mung bean cultivated in four major cropping areas of Pakistan, the results identified bacterial nodule communities dominated by either *Bradyrhizobium* or *Ensifer* strains, depending on the edaphoclimatic conditions (Hakim et al., 2020). *Ensifer* sequences corresponded to 99% of the total nodule rhizobial sequences in mung bean cultivated in a desert soil, while *Bradyrhizobium* sequences amounted to up to 94% under milder conditions. In contrast to data from the studies performed in Pakistan, we did not find OTUs related to *Ensifer* in the present study, suggesting that this genus is either not present or it is not capable of colonizing mung bean cultivated in tropical soil conditions.

An evaluation of nodule microbiome of cowpea cultivated in Brazilian soils also did not detect sequences of the *Ensifer* genus (Leite et al., 2017). However, results from rhizobial isolation using specific cultural medium reveal that cowpea displays a low symbiotic specificity toward the micro-symbiont, and therefore it is able to nodulate with a broad range of different rhizobial species: *Bradyrhizobium*, *Rhizobium*, *Ensifer*, and *Mesorhizobium* (Zhang et al., 2007; Silva et al., 2012; Tulu et al., 2018). The low specificity trait has led cowpea to be used as a trap plant in several studies for isolating rhizobia from soil (Guimarães et al., 2012; Silva et al., 2012; Castro et al., 2017). Controversial results obtained from different techniques are not uncommon and may be either caused by the use of rich nutrient culture media which may favor some bacterial groups, which is nevertheless a minor nodule occupant; or inherent differences due to amplification efficiency of each sequence. This may explain why some rhizobium strains isolated from nodules display poor or no capacity as a micro-symbiont (Zilli et al., 1999; Yang et al., 2008; Zhang et al., 2008; Lu et al., 2009). The lack of *Ensifer* sequences in both cowpea and mung bean nodules analyzed by culture-independent methods suggests that the genus may have limited symbiotic ability under tropical edaphoclimatic conditions.

Presence of *Ensifer* genus from the nodule microbiome of mung bean cultivated in Pakistan may be related to characteristics of local soils. OTUs belonging to the *Ensifer* genus have been found in mung bean nodules grown in alkaline soils with a pH higher than 7.8 (Hakim et al., 2020). Furthermore, the *Ensifer* genus was only found to be dominant in desert soil (Hakim et al., 2020). The soils used in our study have acidic characteristics, with pH between 4.3 and 6.5, and whose place of origin have mean annual precipitation varying from 1,100 to 1,794 mm. Microbial communities are mainly influenced by soil characteristics such as pH (Fierer and Jackson, 2006; Lauber et al., 2009; Rousk et al., 2010; Andrew et al., 2012), temperature (Braker et al., 2010; Garcia-Pichel et al., 2013; Zhou et al., 2016), and rainfall (Chen et al., 2015). The data from both Pakistan and Brazil suggest that the *Ensifer* genus is mainly capable to form symbiosis when mung bean is cultivated in arid alkaline soils.

*Rhizobium* and *Mesorhizobium* strains isolated from mung bean nodules using cultural media were capable of forming nodules under controlled conditions (Yang et al., 2008; Zhang et al., 2008; Lu et al., 2009). However, we did not detect sequences of these genera in our study. A small percentage of sequences belonging to *Rhizobium* and *Mesorhizobium* genera was found in mung bean nodules grown in Pakistan, corresponding to 2.06 and 0.06% for *Rhizobium* and *Mesorhizobium*, respectively (Hakim et al., 2018). In another study, *Mesorhizobium* OTUs were not found, while *Rhizobium* corresponded to only 0.8% of the total sequences analyzed (Hakim et al., 2020). In a study conducted in Venezuela, *Rhizobium* strains have been isolated from mung bean nodules (Ramírez et al., 2020). In this sense, the absence of OTUs from *Ensifer*, *Rhizobium*, and *Mesorhizobium* genera in our study could be related to either a PCR bias caused by the low concentration of these organisms, or a possible allocation of these OTUs as unclassified groups (Lee et al., 2012; Van Dijk et al., 2014). In conclusion, although these genera are able to form nodules in mung bean, they may not be considered as a main micro-symbiont for the crop. It is possible that some of these strains are like a nodule NRB, which nevertheless does not have a clear role.

The *Pseudomonas* genus was the most abundant NRB observed on the nodule bacterial populations evaluated, except for the SE_RJ-II, SE_RJ-III soil samples and Camaleão genotype. These results suggest that bacterial nodule diversity is influenced by the soil and regulated by the plant, which implies that specificity toward the micro-symbionts may be genotype dependent (Hartmann et al., 2009; Wagner et al., 2016; Leite et al., 2017; Liu et al., 2019). Furthermore, *Pseudomonas* sequences may have arised from Esmeralda seeds, but since this has been observed in eight out of 10 soil samples, we might consider that other intrinsic factors are influencing the pattern. Plant tissue endophytes may originate either from environmental infection (horizontal transmission), or be vertically transmitted via seed or vegetative propagation (Johnston-Monje and Raizada, 2011; Yan et al., 2019). Legume nodule colonization by symbiotic bacteria is a consequence of a complex genetic mechanism which has been well described, while colonization mechanisms by NRB are still unclear, although it appears the plant plays an important role (Wang et al., 2018; Gage, 2020). In comparing plant and soil origin influences on nodule colonization by NRB, plant genotypes possess more favorable traits related to nodule occupancy by microbial communities (Muresu et al., 2008; Pandya et al., 2013; Zgadzaj et al., 2015; Regus et al., 2017; Westhoek et al., 2017; Sachs et al., 2018). Nodulation factors involved in plant-microorganism chemotaxis may lead to a selection of associated organisms (Kobayashi and Broughton, 2008; Wang et al., 2012). *Pseudomonas* strains have already been found in mung bean nodules grown in Pakistan (Hakim et al., 2018, 2020), as well as in other legume species (Kuklinsky-Sobral et al., 2004; Hoque et al., 2011; Aserse et al., 2013; Oliveira-Longatti et al., 2014; De Meyer et al., 2015; Leite et al., 2017; Cardoso et al., 2018).

In addition to *Pseudomonas*, another 16 NRB genera were shown by in our unfiltered data (Supplementary Table S1). Except for *Pseudomonas* strains, the presence of NRB genera sequences does not seem to be important, considering the low numbers and the inconsistency among replicates. The under representativeness of these taxa may be related to a low soil bacterial abundance or to inherent difficulties during the amplification reaction by the NGS method.

In summary, the knowledge acquired from our results should support the development of new inoculants for mung bean under tropical condition. To this end, two premises were used: soil samples from agriculture areas with a legume cultivation history were used as a seed inoculant; and, conditions where plant were grown would favor BNF, consisting of a reduced amount of soil and a nutrient solution devoid of N. The dominance of *Bradyrhizobium* strains inhabiting mung bean nodules cultivated on different soil samples, regardless of plant genotype, suggests that this is the main nodule micro-symbiont for the crop in Brazilian tropical soil areas evaluated. Additionally, the bacterial communities showed the ability to form reddish nodules, which is an indicative of the presence of leghemoglobin and an active nitrogenase (Ott et al., 2005; Singh and Varma, 2017; Larrainzar et al., 2020).

The bacterial community strategy used in our study was capable to provide the identification of a pattern that may guide the development of a new rhizobial inoculant for mung bean capable of increasing BNF and grain yield. Technological implementation will require isolating and selecting efficient strains for the crop, which will be our next step continuing this work. In addition, co-inoculation with *Pseudomonas* strains will also be evaluated. A greater richness of beneficial microorganisms colonizing nodules is thought to contribute to plant growth, as well as to improve resistance to pathogens. From the results, we suggest that an efficient *Bradyrhizobium* strain on its own or co-inoculated with *Pseudomonas* strains, in this case, dependent on the plant genotype, could promote mung bean growth and improve grain yield, thereby resulting in a better cost/benefit ratio taking into account the agriculture production.

## Data Availability Statement

The datasets presented in this study can be found in online repositories. The names of the repository/repositories and accession number(s) can be found below: NCBI (accession: PRJNA629841).

## Author Contributions

VF, RC, VM, and AL contributed to soil sample collection, experiment installation, and plant collection. VF, MC, and NR contributed to DNA extraction and bioinformatics analysis. VF, MC, NR, GX, and SU contributed to the writing and revision of the manuscript. All authors read, edited, and approved the final manuscript.

## Conflict of Interest

The authors declare that the research was conducted in the absence of any commercial or financial relationships that could be construed as a potential conflict of interest.

## Supplementary Material

Supplementary Figure 1 Diagram of adapted Leonard jars with the three layers: substrate mixture (gravel and vermiculite), soil sample and sand.

Supplementary Table 1 Illumina MiSeq sequence raw data from Mothur software.
