# Supplementary figures and images for: Bradyrhizobium as the Only Rhizobial Inhabitant of Mung Bean (Vigna radiata) Nodules in Tropical Soils: A Strategy Based on Microbiome for Improving Biological Nitrogen Fixation Using Bio-Products
Source: Front Plant Sci. 2021 Jan 12;11:602645. doi: 10.3389/fpls.2020.602645 (PMC7835340; doi:10.3389/fpls.2020.602645)

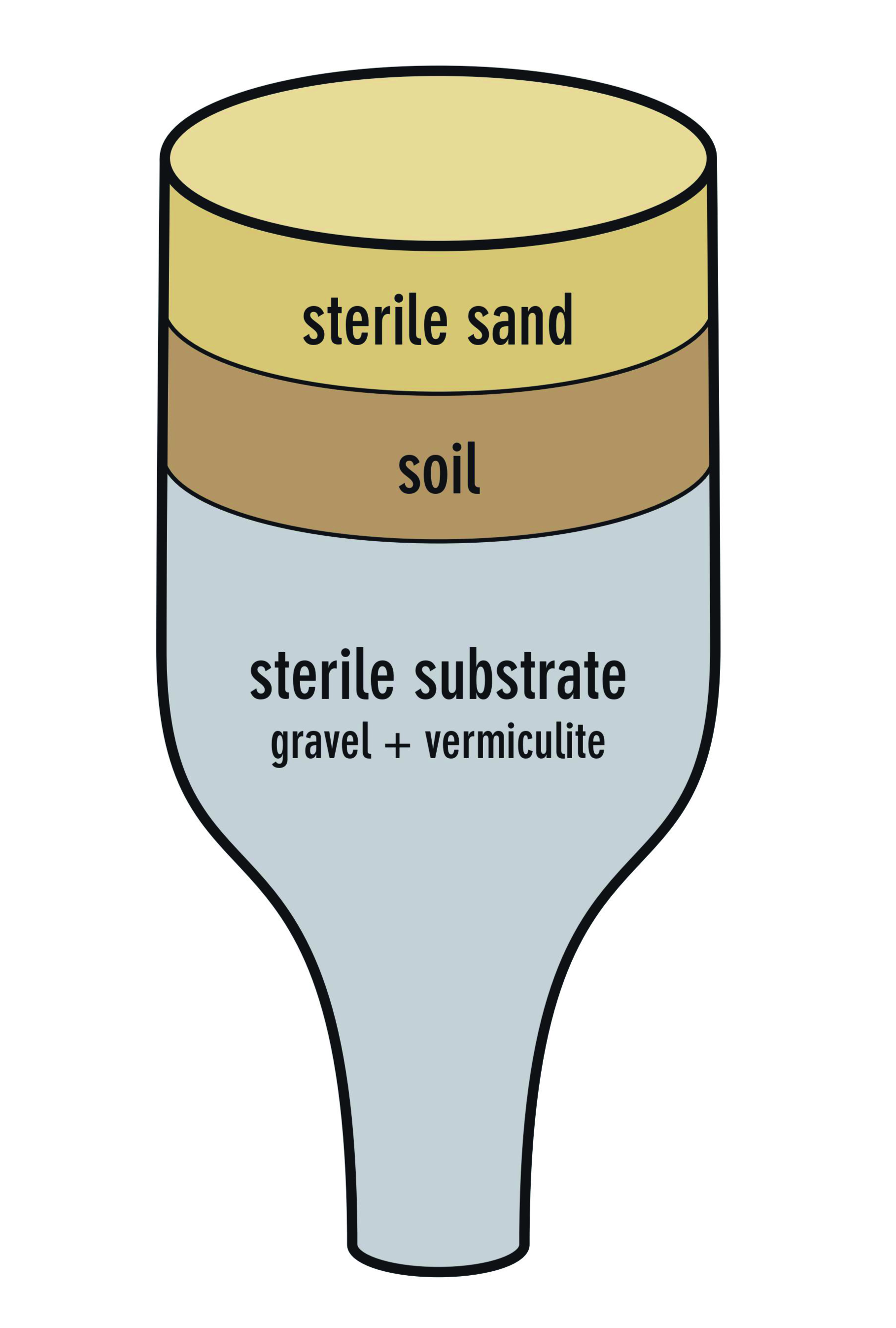

Supplement: Supplementary Figure 1 — Diagram of adapted Leonard jars with the three layers: substrate mixture (gravel and vermiculite), soil sample and sand. [file Image_1.TIF]
